# Microsatellite markers reveal genetic diversity and population structure of *Portunus trituberculatus* in the Bohai Sea, China

**DOI:** 10.1038/s41598-023-35902-1

**Published:** 2023-05-29

**Authors:** Baohua Duan, Tongxu Kang, Haifu Wan, Weibiao Liu, Fenghao Zhang, Shumei Mu, Yueqiang Guan, Zejian Li, Yang Tian, Xianjiang Kang

**Affiliations:** 1 College of Life Sciences, Hebei University, Baoding, 071000 China; 2 Bureau of Agricultural and Rural Affairs of Huanghua City, Huanghua, 061100 China; 3 Hebei Fishery Technology Extension Station, Shijiazhuang, 050000 China; 4 Institute of Life Science and Green Development, Hebei University, Baoding, 071000 China; 5 Hebei Province Innovation Center for Bioengineering and Biotechnology, Baoding, 071000 China

**Keywords:** Genetics, Ocean sciences

## Abstract

The swimming crab, *Portunus trituberculatus*, is one of the main aquaculture species in Chinese coastal regions due to its palatability and high economic value. To obtain a better understanding of the genetic diversity of *P. trituberculatus* in the Bohai Sea, the present study used 40 SSR loci to investigate the genetic diversity and population structure of 420 *P. trituberculatus* individuals collected from seven populations in the Bohai Sea. Genetic parameters revealed a low level of genetic diversity in the cultured population (*SI* = 1.374, *He* = 0.687, and *PIC* = 0.643) in comparison with wild populations (*SI* ≥ 1.399, *He* ≥ 0.692, and *PIC* ≥ 0.651). The genetic differentiation index (*Fst*) and gene flow (*Nm*) ranged from 0.001 to 0.060 (mean: 0.022) and 3.917 to 249.750 (mean: 31.289) respectively, showing a low differentiation among the seven populations of *P. trituberculatus*. Population structure analysis, phylogenetic tree, and principal component analysis (PCA) demonstrated that the seven groups of *P. trituberculatus* were divided into four subpopulations (K = 4), but the correlation between genetic structure and geographical distribution was not obvious. These results are expected to provide useful information for the fishery management of wild swimming crabs.

The swimming crab, *Portunus trituberculatus*, is one of the important economic crabs in the Chinese marine fisheries and mariculture industry. It has a wide distribution in the coastal areas of South-East Asia and has been farmed for more than 30 years^1–3^. Over the past few decades, the consumption of swimming crab has gradually increased due to the delicious taste and versatile nutrients^4^. Among the main producers, China ranked first with an annual production of 559,796 tons according to the China Fisheries Statistical Yearbook (2022) published by the Ministry of Agriculture, China. However, with the development of intensive farming and marine fishing industry in recent years, germplasm resources of *P. trituberculatus* have dramatically declined due to over-exploitation and environmental deterioration^5,6^. In addition, the heavy demand for wild parents from artificial propagation resulted in the decline of the genetic diversity of the natural populations^7^. Such episodes emphasize the vital nature of monitoring the genetic diversity of *P. trituberculatus* populations to protect germplasm resources and facilitate molecular marker-assisted breeding (MAS).

Investigating the genetic diversity of species is a prerequisite for the effective exploration and utilization of germplasms^8^. A high level of genetic diversity indicates strong biological survivability and environmental adaptation, which is required for sustained genetic improvement and stable inheritance of desirable traits^9^. Conversely, low genetic diversity can lead to reduced adaptability and viability, and ultimately to the degradation of species^10^. In aquaculture, genetic diversity constitutes a fundamental resource to improve the quality of stock^11^. However, for breeding populations of *P. trituberculatus*, long-term artificial directional selection eventually leads to a decline in genetic diversity^12^. Moreover, it is difficult to recover the declining genetic diversity caused by overfishing^13^. To formulate an effective conservation strategy, it is necessary to evaluate the genetic diversity and population structure of *P. trituberculatus*. In our previous study, SNP markers determined by genotyping-by-sequencing (GBS) revealed a low level of genetic diversity in *P. trituberculatus* along the coastal waters of China^14^. To evaluate the impact of the massive releases on natural populations, the researchers monitored the temporal variations in genetic diversity and structure in Panjin and Yingkou using microsatellite markers, which suggested that the large-scale stock enhancement of *P. trituberculatus* presented potential genetic risks to wild populations^15,16^. However, hatchery stock enhancements resulted in no reduction in genetic diversity for wild populations of *P. trituberculatus* in the Yangtze Estuary^17^.

The development of high-throughput sequencing technologies provides great convenience for the identification of DNA molecular markers in genetic research. Among known DNA molecular markers, simple sequence repeat (SSR) shows the advantages of co-dominant inheritance, highly polymorphic, and wide distribution throughout the genome^18–20^. At present, RNA-seq has become a popular high-throughput sequencing technology that enables the development of SSR markers due to its characteristics of wide dynamic range, high accuracy, and strong sensitivity^21^. In addition, compared with genomic-derived SSRs, transcriptome-derived SSRs are characterized by high efficiency, strong transferability, and correlation with potential genes^22^. Cao et al.^23^ first analyzed the transcriptome of *Crassadoma gigantean* using RNA-seq technology, identified 12 polymorphic SSRs, and found several genes related to the growth and immunity of *C. gigantean*. These results would facilitate future studies of population structure and conservation genetics in this species. In aquatic crustaceans, Zhang et al.^24^ conducted transcriptome sequencing on the male and female gonads of *Portunus sanguinolentus* and detected 93,196 SSR loci. In *Pachygrapsus marmoratus*, 43,915 SSRs were excavated by RNA-seq, providing a reliable resource for investigating biological responses to pollution in intertidal and marine populations^25^. Lv et al.^6^ identified 22,673 SSRs with transcriptome analysis of *P. trituberculatus*, which provided a material basis for genetic linkage and quantitative trait loci analyses. The objective of the current study is to evaluate the genetic diversity and population structure of *P. trituberculatus* in the Bohai Sea with transcriptomic SSRs*.* The findings will contribute to understanding the population genetic structure of *P. trituberculatus* in the Bohai Sea and be useful in improving management and conservation strategies for this species.

## Material and methods

### Sample collection and DNA extraction

**Figure 1:**
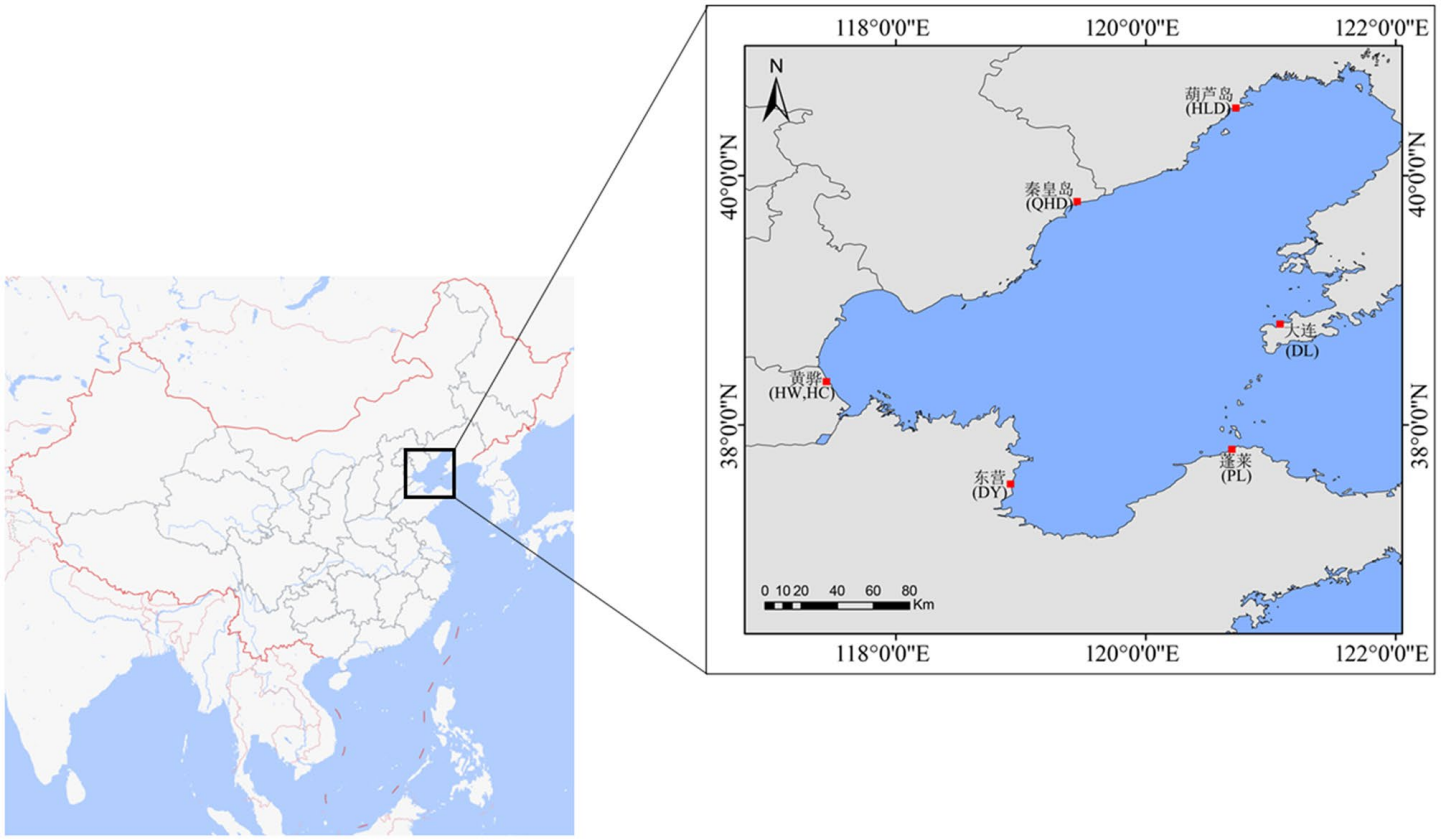
Swimming crab sampling locations. Note: This figure was created by DIVA-GIS 7.5 software (http://swww.diva-gis.org/).

A total of seven populations were collected from the Bohai Sea (Fig. 1, Table 1). Six wild populations included Dalian (DL), Huludao (HLD), Qinhuangdao (QHD), Huanghua (HW), Dongying (DY), and Penglai (PL). One cultured population (HC) that was sampled from the national breeding farm of swimming crabs in Huanghua (Hebei, China) came from the Bohai Sea. The claws of all individuals were collected and immediately preserved in 95% ethanol and stored at −20 °C. Genomic DNA was isolated from claw muscle using the TIANamp Marine animal DNA extraction kit (TIANGEN, Beijing, China) following the manufacturer's recommended protocols. After extraction, the quality and concentration of DNA samples were determined using a NanoDrop2000 spectrophotometer (Thermo Fischer Scientific), quantified, diluted to 100 ng/μl, and stored at s−20 °C.

**Table 1:** Sampling information of seven *P. trituberculatus* populations from the Bohai Sea.

Population	Abbreviation	Number	Longitude (E°)	Latitude (N°)
Dalian	DL	60	121°53′28″	39°05′01″
Huludao	HLD	60	120°84′40″	40°72′94″
Qinhuangdao	QHD	60	119°60′22″	39°95′52″
Huanghua (wild)	HW	60	117°64′22″	38°49′21″
Dongying	DY	60	118°93′99″	37°49′10″
Penglai	PL	60	120°75′66″	37°83′29″
Huanghua (cultured)	HC	60	117°64′09″	38°48′99″

### PCR amplification and capillary electrophoresis

Forty pairs of SSR primers were obtained from the transcriptome data in our previous study^26^ (Table 2). All forward primers were labeled with the fluorescent dye, 6-carboxy-fluorescein (FAM). Polymerase chain reaction (PCR) amplification was performed in 20 µL reaction volumes containing 2 μL of template DNA, 2 μL of each primer (2.5 μmol/L each), 10 μL of 2 × Es Taq Master Mix (CWBIO, Beijing, China) and 4 μL of ddH_2_O. Amplification cycles consisted of initial denaturation (5 min at 95℃), followed by 35 cycles of denaturation (30 s at 94 ℃), annealing (30 s), extension (30 s at 72 °C) and additional extension (10 min at 72 °C). After amplification, PCR products were diluted 10 times with sterile water. The pooled sample was composed of 20 μL Hi-Di formamide and 0.2 μL GeneScan 500 ROX size standard. An ABI 3730XL Genetic Analyzer (Applied Biosystems, Foster City, CA) was used to conduct capillary electrophoresis (CE) following the manufacturer's instructions. Each CE sample contained 1μL diluted PCR product and 15 μL pooled sample. Allele sizes (in base pairs) were determined with GeneMarker®Fragment Analysis Software (Softgenetics LLC®, State College, PA, USA) on the comparison of the position of the internal size standard in each lane with the position of the peak value of each sample.

**Table 2:** Characteristics of 40 SSR loci for *P. trituberculatus*.

Loci	Primer sequence (5′ to 3′ )	Repeat motif	Product size (bp)	Tm (℃)
TRAN1	F:CTACCGGAGTTTTCGAAGGTAAC	(AGG)_8_	140–165	60
	R:GATCACGGGAAAGAGTTGCTAT			
TRAN2	F:TCACTACCACTACCGCTTTGTTT	(CAC)_8_	125–155	60
	R:GATGTCAGTAACGGGAGAGTGAG			
TRAN3	F:GCTGTTGTAGAAACCCATGAAAG	(GTG)_7_	110–140	60
	R:AGGGAGATACACGACCAACACTA			
TRAN4	F:CTCCTCCCCAGTGTTCTCTATTT	(CCT)_9_	95–125	60
	R:GACAATAACGATGACGACAGTGA			
TRAN5	F:CTCCGTGTTGGCTATTAGCTTTA	(ACC)_9_	125–170	60
	R:TGTGTGCTGTTAGCGTATATTGG			
TRAN6	F:GCGTTACCGTTACCACTATGAAG	(TGG)_10_	90–125	60
	R:CATCACATCCTTCTTATCCTTCG			
TRAN7	F:CACGATCGTAGAAGAAAAGTTGG	(GTG)_9_	90–130	60
	R:CCTTCTCTTCCTCCTCTTGTTTC			
TRAN8	F:AGTGAGTTGCTTCCACTTCTGTC	(GAG)_11_	110–147	60
	R:CTATTGTAAGCATCCCTCCTCCT			
TRAN9	F:GTTCAGAAGGTCTGCGAGATAAA	(CAC)_6_	105–145	60
	R:GCTAAAACTTCACTCATTGGTGG			
TRAN10	F:TCGTCCTCTTTCTCCTCTCTTTT	(CTC)_7_	125–150	60
	R:ACAATACTTATTTGTGGGGAGGG			
TRAN11	F:GCTGTGAGTTTCACTTGTTTGTG	(CA)_16_	170–238	60
	R:GCTGCCTACAGTCTTGTCTCTTC			
TRAN12	F:CGGGAACCTTAGCGTTAAGTAGT	(GT)_14_	147–217	60
	R:TATATCTATTGCGCACCTCACCT			
TRAN13	F:GTGACAGTGTCCCTACCTTCTTG	(TG)_9_	145–180	60
	R:TCTACCATGGTCTCCAAGTTTGT			
TRAN14	F:GCTTCCTTACCCTAAGCAGAAAC	(GT)_9_	125–180	60
	R:ATGTATGTCAGTCGGAGACCATC			
TRAN15	F:TCAGCTGTAAGTCTGAAAGTCCC	(CA)_10_	140–200	60
	R:CAGCTAGTTCAGGAATTAAGGCA			
TRAN16	F:TCCTGCTTTCCAACTTCTCTATG	(TG)_10_	110–147	60
	R:CCCTCCCGTAAAATACAACTAGG			
TRAN17	F:TTACTGGGTAGAAGTCCGTACCA	(AC)_8_	123–165	60
	R:TGATAGGGCTATAGAGAGCAACG			
TRAN18	F:GCGTAAATCTGCTCGTCTGTACT	(TG)_8_	110–145	60
	R:TCTCTCTCTCGAATGATGTGTCA			
TRAN19	F:ATTATCACCAGGGATGTCAGGA	(AC)_15_	170–217	60
	R:AGTGACTGTGGGTTTTGTTGTCT			
TRAN20	F:CAGCACAGAATGTAAGGATGTGA	(GT)_16_	130–180	60
	R:CCTTACTTGAATCTGTACCCACG			
TRAN21	F:AGCTTTGTGACAGACATGGAACT	(GT)_13_	147–200	60
	R:CCATTAGCTTCCTATCACCCTCT			
TRAN22	F:TAAAGCCAGCGCTCTAACTACTG	(GT)_10_	100–145	60
	R:AGGTCACTACTGGGTGGCACTAT			
TRAN23	F:GAAGTGACTAACCGAGCGTACAT	(TG)_12_	130–190	60
	R:CAGCCATAAACACCCTCTAAATG			
DX05	F:GTGGGCCGCCAATATCACTA	(TG)_12_	140–180	60
	R:AATCCACCACTTGCACCCAA			
DX07	F:CGTGCATCCGTGTGTTTGTT	(TG)_10_	115–155	60
	R:GCCATCTTTTCGCCGAGTTG			
DX09	F:TAGGCATGGGATGGGTGAGA	(CA)_17_	140–200	60
	R:CGGGAAGGAGTGTTGTTGAGT			
DX10	F:AATCACAACCCAGCCGCATA	(TG)_12_	110–147	60
	R:ACAACGAAGGAGAGATGCGG			
DX14	F:CCCGCTACCCCATAACTCAC	(GTG)_7_	120–175	60
	R:TCTTCCTCCCCACAGCCATA			
DX15	F:CGTCCCATCATCTGACAAAGG	(GAG)_6_	200–240	60
	R:TCCTTCACCTCTTCCTCTTTTCT			
DX16	F:GAGGCAAGCAAGTTAACCATTAG	(GT)_7_	110–147	60
	R:CTTCCTGGTTACCTCATCCTACC			
DX19	F:CACACTCGTTGCAGACACTACTT	(TG)_11_	160–217	60
	R:CTGTTACTTACTCGGTGCTTTGG			
ZL05	F:AGAATGTTGCCATGGCTGGA	(GGT)_7_	160–180	60
	R:ACCCTGTATCAGTGCGTTGG			
ZL06	F:CCCGCCCCTGTACATTTTCA	(TAT)_10_	135–180	60
	R:TGTTGGTAGGCTTGGTGGTC			
ZL08	F:GCTTCTGCTGCTGGTCCTTA	(CAAC)_10_	110–130	60
	R:ACCAGACATTGCTGAGCATG			
PrMa01	F:CCTTGCCTCGTCAGTGTCAT	(CTG)_6_	123–160	60
	R:TGGCTGTAGACACCCTCCAT			
PrMa02	F:AGAGCTGACCTCGCTTTGAC	(GTG)_8_	160–190	60
	R:TCCAGCTCCTCCTGTCCAAT			
PrMa03	F:CTTGATTGCCTCTCGCTTGT	(TG)_10_	147–201	60
	R:GGGGGAGAGGGAGAGAATGT			
PrMa04	F:TCCTGGACCTTGTTCAGTCC	(TCC)_10_	123–155	60
	R:GCAATCCCACACACACTCCT			
PrMa05	F:GCGTTGCGTGTACTGAAAGT	(TG)_31_	190–242	60
	R:GCGGCTCTGGTCAGGAATAC			
PrMa06	F:TCCTGCAACTTACATTCTTGGTC	(CA)_15_	160–201	60
	R:GTGTGCACAGGATACAGCCT			

### Data analysis

Genetic diversity within *P. trituberculatus* populations was estimated by determining genetic parameters, including the number of alleles (*Na*), the effective number of alleles (*Ne*), Shannon’s diversity index (*SI*), observed heterozygosity (*Ho*) and expected heterozygosity (*He*) using POPGENE version 1.3^27^. Based on allele frequency, polymorphism information content (*PIC*) was estimated by PIC-CALC software^28^. Null allele frequencies (*F*na) for SSR loci were calculated using GenePOP^29^. *P* values were calculated for determining Hardy–Weinberg equilibrium (HWE) at each locus with POPGENE version 1.3. Genetic differentiation and variation were inferred using Nei's genetic distance (*D*)^30^ and genetic identity (*I*) calculated by POPGENE version 1.3 and F-statistics (*Fst*, *Fis*) calculated by analysis of molecular variance (AMOVA) with software GenAlEx 6.5^31^ through 999 permutations. Gene flow (*Nm*) was inferred from the formula of *Nm* = (1 − *Fst*)/4*Fst*^32^.

The phylogenetic tree was constructed based on Nei’s genetic distance and used to test population grouping as implemented in MEGA7^33^. Principal component analysis (PCA) was carried out using Canoco 4.5 to elucidate genetic relationships within and among *P. trituberculatus* populations. Based on the 40 polymorphic SSR loci, Bayesian model-based population genetic structure was inferred using STRUCTURE version 2.3.4^34^. The putative number of populations (K) was set from 1 to 10 with 3 replicate simulations for each K value using 100,000 MCMC (Markov Chain Monte Carlo) iterations after an initial 100,000 burn-in period. With the log probability of data (LnP(D)) and an ad hoc statistic ΔK based on the rate of change in LnP(D) between successive K-values, the structure output was entered into Structure Harvester^35,36^ to determine the optimum K value. The best K value was analyzed by CLUMPP^37^ and visualized with Distruct 1.1 software^38^.

## Results

### Genetic diversity within populations

In this study, all parameters of the 40 SSR loci were calculated and presented in Table 3. A total of 217 alleles were found with an average of 5.425 per locus. The effective number of alleles (*Ne*) ranged from 1.785 to 10.271 with a mean of 4.264. Shannon’s diversity index (*SI*), observed heterozygosity (*Ho*) and expected heterozygosity (*He*) ranged from 0.885 to 2.404 (mean: 1.482), 0.405 to 0.950 (mean: 0.639) and 0.440 to 0.903 (mean: 0.725), respectively. *PIC* values ranged from 0.415 (TRAN1) to 0.895 (TRAN20) with an average of 0.685. Five SSRs (TRAN1, TRAN3, ZL05, DX14, and TRAN13) showed moderate polymorphism (0.25 < *PIC* < 0.5), and the remaining 35 SSRs showed high polymorphism (*PIC* > 0.5). Null allele frequencies (*F*na) and fixation index (*Fis*) varied from 0.029 (DX19) to 0.564 (TRAN13) and -0.207 (DX19) to 0.478 (TRAN21) respectively, indicating the existence of null alleles and heterozygosity deficit. Additionally, nine SSR loci fitted with HWE (*P* > 0.05), and the remaining 7 and 24 loci deviated from HWE at *P* < 0.05 and* P* < 0.01 levels, respectively.

**Table 3:** Genetic parameters for 40 SSR loci.

Loci	*Na*	*Ne*	*SI*	*Ho*	*He*	*PIC*	*F*na	*Fis*	*P*
TRAN1	5	1.785	0.911	0.452	0.44	0.415	0.085	−0.041	NS
TRAN2	4	2.725	1.148	0.633	0.633	0.575	0.051	−0.01	*
TRAN3	4	1.829	0.895	0.414	0.453	0.425	0.135	0.062	*
TRAN4	4	3.302	1.277	0.702	0.697	0.642	0.139	−0.017	NS
TRAN5	4	2.774	1.150	0.531	0.640	0.578	0.537	0.161	**
TRAN6	4	3.095	1.242	0.633	0.677	0.624	0.480	0.054	*
TRAN7	5	3.621	1.417	0.662	0.724	0.678	0.107	0.08	**
TRAN8	6	3.436	1.504	0.700	0.709	0.680	0.123	0.001	*
TRAN9	5	4.008	1.495	0.510	0.751	0.713	0.243	0.316	**
TRAN10	4	2.597	1.123	0.524	0.615	0.557	0.177	0.14	**
TRAN11	6	4.481	1.630	0.679	0.777	0.744	0.187	0.118	**
TRAN12	6	5.164	1.716	0.512	0.806	0.779	0.253	0.349	**
TRAN13	3	2.275	0.921	0.557	0.561	0.480	0.564	−0.001	NS
TRAN14	3	2.766	1.058	0.629	0.638	0.566	0.290	0.004	*
TRAN15	6	5.303	1.723	0.798	0.811	0.784	0.074	0.009	NS
TRAN16	4	2.373	1.082	0.595	0.579	0.532	0.194	−0.046	**
TRAN17	5	3.453	1.420	0.576	0.710	0.674	0.156	0.180	**
TRAN18	5	4.487	1.554	0.771	0.777	0.742	0.115	−0.004	NS
TRAN19	8	7.236	2.031	0.721	0.862	0.846	0.178	0.155	**
TRAN20	12	10.271	2.404	0.817	0.903	0.895	0.168	0.088	**
TRAN21	7	5.896	1.863	0.429	0.830	0.810	0.347	0.478	**
TRAN22	5	4.086	1.490	0.681	0.755	0.715	0.372	0.083	*
TRAN23	11	8.670	2.277	0.826	0.885	0.874	0.205	0.054	**
DX05	8	5.890	1.925	0.857	0.830	0.810	0.080	−0.065	NS
DX07	4	3.019	1.217	0.645	0.669	0.608	0.128	0.024	NS
DX09	6	5.214	1.721	0.671	0.808	0.782	0.251	0.153	**
DX10	4	3.482	1.315	0.679	0.713	0.663	0.171	0.035	**
DX14	3	2.099	0.886	0.531	0.524	0.460	0.252	−0.024	NS
DX15	4	3.947	1.379	0.405	0.747	0.699	0.369	0.439	**
DX16	5	3.725	1.421	0.714	0.732	0.685	0.081	0.012	NS
DX19	6	4.808	1.666	0.950	0.792	0.761	0.029	−0.207	**
ZL05	3	2.253	0.885	0.429	0.556	0.458	0.155	0.180	**
ZL06	7	5.501	1.817	0.691	0.818	0.794	0.236	0.018	**
ZL08	6	4.725	1.675	0.802	0.788	0.760	0.098	−0.029	*
PrMa01	6	5.037	1.709	0.836	0.802	0.775	0.134	−0.099	**
PrMa02	5	3.849	1.450	0.533	0.740	0.698	0.480	0.204	**
PrMa03	7	6.105	1.873	0.638	0.836	0.815	0.330	0.151	**
PrMa04	5	4.342	1.534	0.641	0.770	0.733	0.190	0.029	**
PrMa05	6	5.424	1.739	0.717	0.816	0.790	0.284	0.105	**
PrMa06	6	5.524	1.750	0.455	0.819	0.794	0.346	0.435	**
Mean	5.425	4.264	1.482	0.639	0.725	0.685	0.220	0.113	–

*Na* Number of alleles; *Ne* Number of effective alleles; *SI* Shannon’s diversity index; *Ho* Observed heterozygosity; *He* Expected heterozygosity; *PIC* Polymorphism information content *F*na Frequency of null alleles; *Fis* fixation index; *P* Probability of significant deviation from Hardy–Weinberg equilibrium; *NS* not significant (*P* > 0.05).

*P < 0.05, **P < 0.01.

The mean values of *Na*, *Ne*, *SI*, *Ho*, *He*, and *PIC* of seven *P. trituberculatus* populations ranged from 5.225 to 5.375, 3.794 to 4.103, 1.374 to 1.449, 0.624 to 0.654, 0.687 to 0.714, and 0.643 to 0.673, respectively (Table 4), revealing a relatively low level of genetic diversity in the cultured population (*SI* = 1.374, *He* = 0.687, and *PIC* = 0.643) in comparison with wild populations (*SI* ≥ 1.399, *He* ≥ 0.692, and *PIC* ≥ 0.651).

**Table 4:** Genetic diversity indices of seven populations of *P. trituberculatus* from the Bohai Sea.

Population	*Na*	*Ne*	*SI*	*Ho*	*He*	*PIC*
DL	5.325	3.959	1.427	0.633	0.709	0.666
DY	5.375	3.884	1.399	0.624	0.692	0.651
HW	5.35	4.038	1.439	0.629	0.713	0.672
HC	5.225	3.794	1.374	0.649	0.687	0.643
HLD	5.375	4.103	1.449	0.654	0.714	0.673
PL	5.35	4.012	1.439	0.641	0.714	0.672
QHD	5.325	3.988	1.427	0.64	0.709	0.667

### Population genetic structure

**Figure 2:**
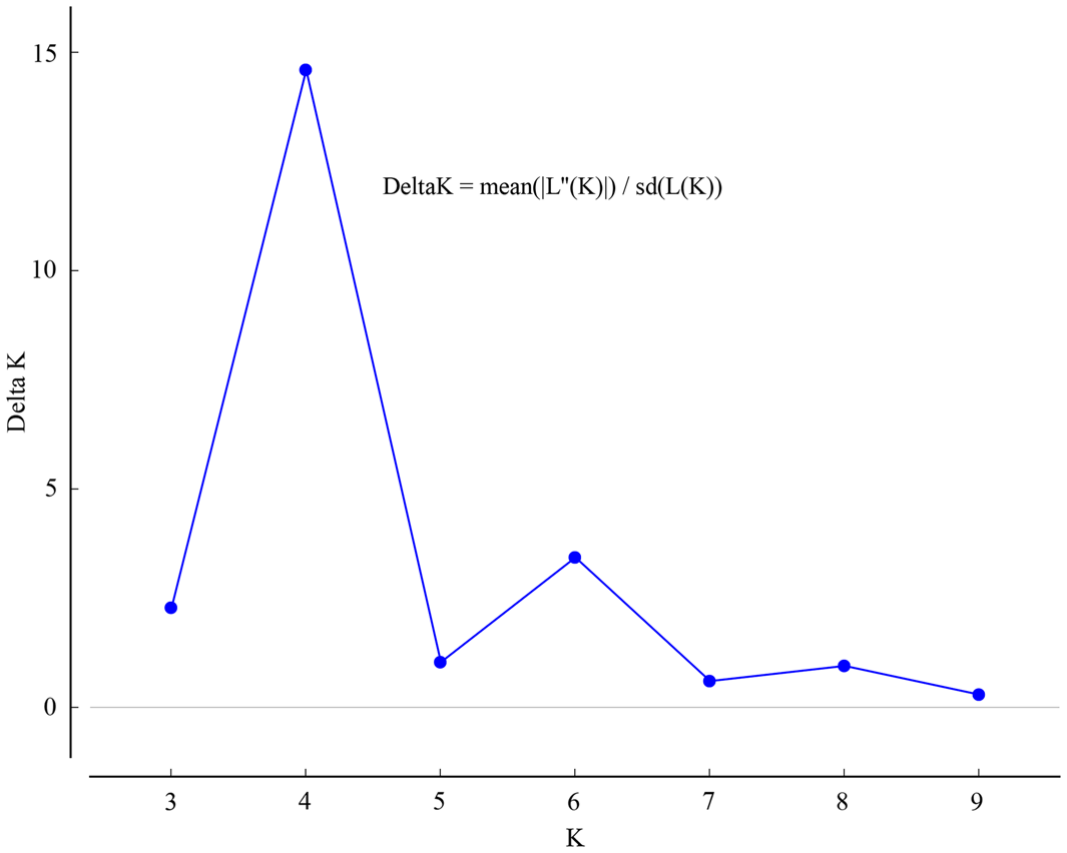
Relationships between the number of clusters (K) and the corresponding Delta K statistics from structure analysis.

**Figure 3:**
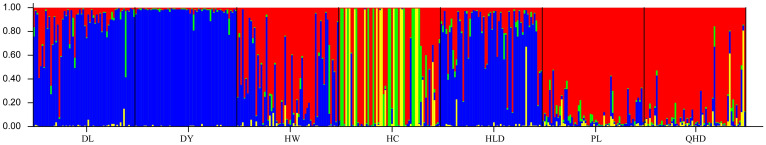
Population genetic structure based on the Bayesian clustering model among 420 *P. trituberculatus* individuals at K = 4.

**Figure 4:**
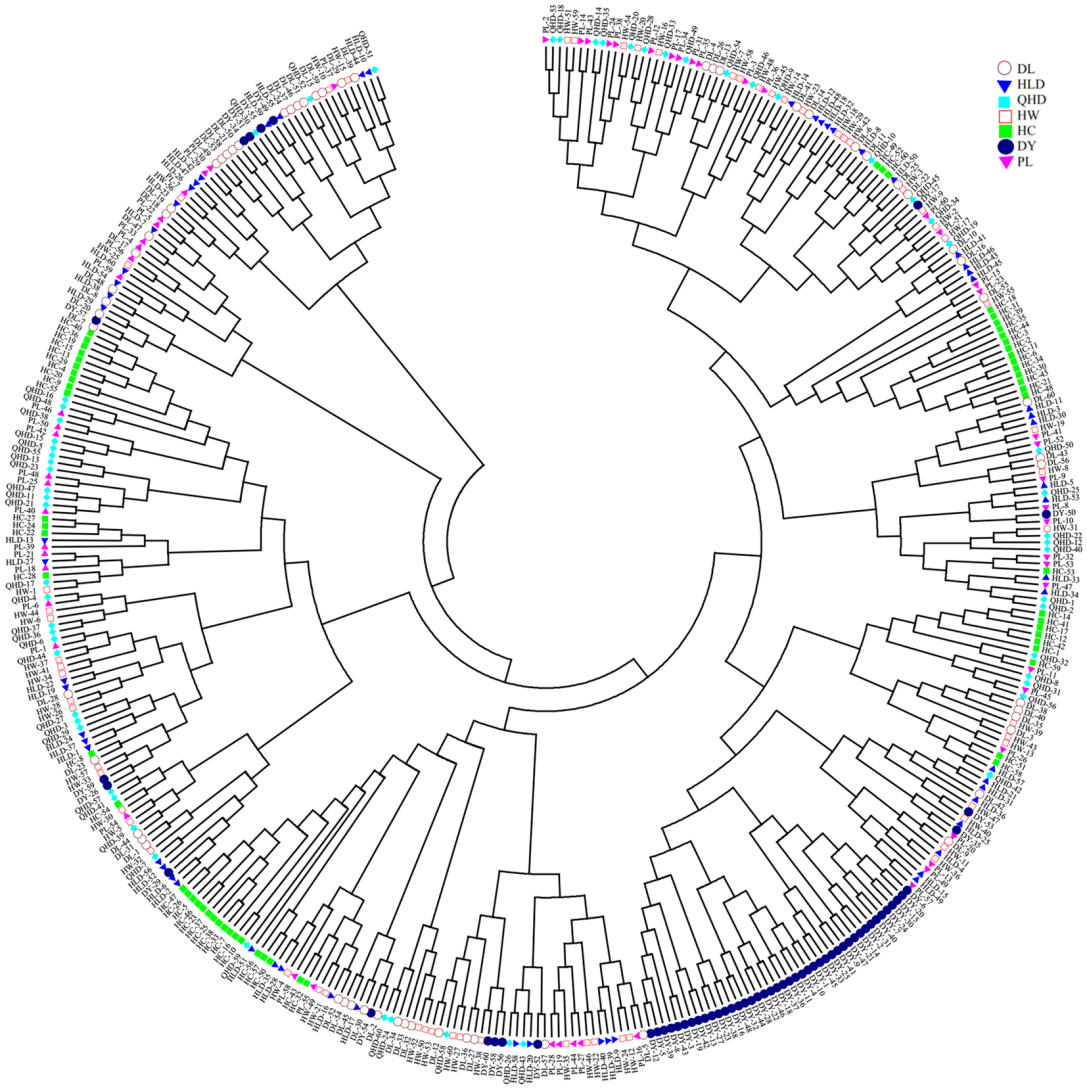
The phylogenetic tree based on Nei's unbiased genetic distance (Nei, 1978) among 420 *P. trituberculatus* individuals.

Genetic structural analysis of the total 420 *P. trituberculatus* individuals was performed to infer the optimal K value with the ΔK method. When the highest ΔK value was observed, the optimal K value was 4 (Fig. 2), which indicated that the seven populations were divided into four subpopulations (Fig. 3). The populations of Dalian (DL), Dongying (DY), and Huludao (HLD) formed a subpopulation (blue). Similarly, the populations of Huanghua (HW), Penglai (PL), and Qinhuangdao (QHD) formed another subpopulation (red). In the cultured population (HC), the genetic components of most individuals were homozygous but formed two subpopulations (green and yellow). The phylogenetic tree at the individual level based on Nei's genetic distances provided supplementary evidence that the HC population was scattered in different branches and DY individuals showed group clustering (Fig. 4).

**Figure 5:**
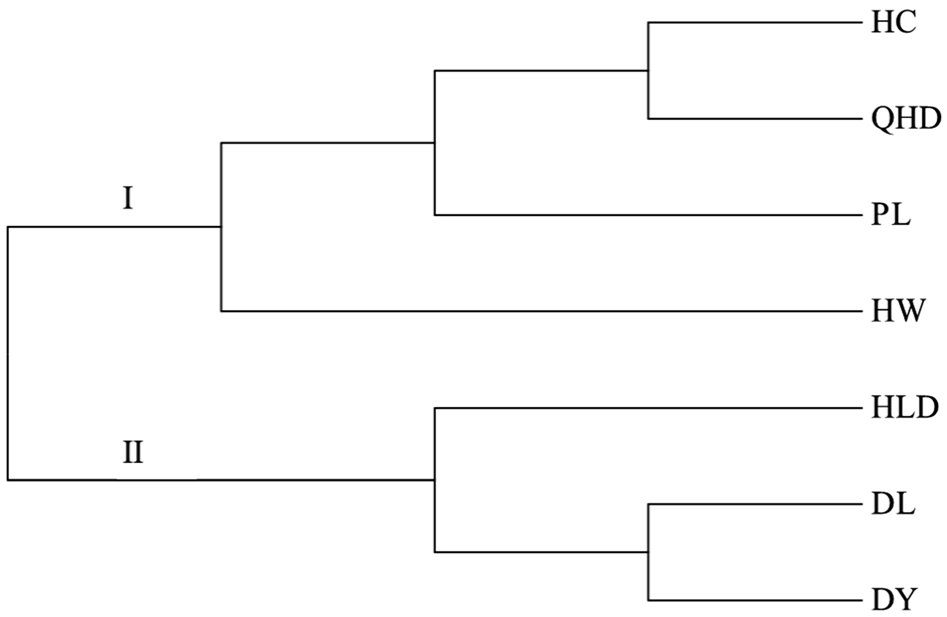
The phylogenetic tree based on Nei's unbiased genetic distance (Nei, 1978) among seven *P. trituberculatus* populations.

**Figure 6:**
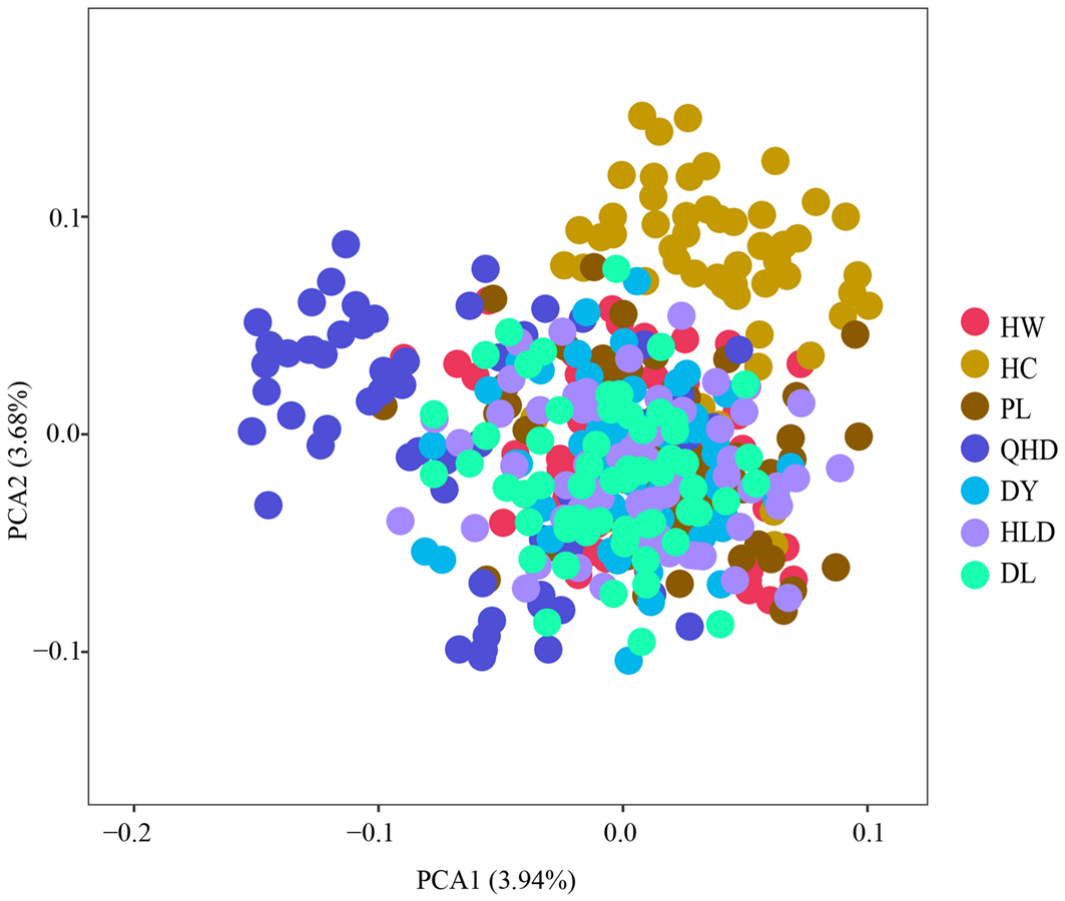
Genetic relationships of 420 *P. trituberculatus* individuals as revealed by principal component analysis (PCA) with 40 SSR loci.

The population clustering results showed that the seven populations of *Portunus trituberculatus* formed two main groups (Fig. 5). Group I included four populations: HC, QHD, PL, and HW. The HC and QHD populations aggregated first, then with PL populations, and finally with HW population. Group II included three populations of HLD, DL, and DY. Overall, DY and HC had the largest genetic distance, which revealed that the genetic structure of *P. trituberculatu*s populations in the Bohai Sea was not significantly related to their geographical distribution. In addition, PCA analysis demonstrated that the first two principal components explained 3.94% (PC1) and 3.68% (PC2) of total variation and could distinguish cultivated individuals from wild populations (Fig. 6). In summary, no obvious geographical distribution pattern was found, which illustrated high genetic mixing and gene flow between individuals of different populations.

### Population differentiation and variation

The low differentiation (*Fst* = 0.001) and high gene flow (*Nm* = 249.750) were observed between the PL and QHD populations, and the high differentiation (*Fst* = 0.060) and low gene flow (*Nm* = 3.917) was observed between the HC and DY populations (Table 5). In addition, Nei's genetic distance (*D*) and genetic identity (*I*) showed similar results between HC and DY populations (*D* = 0.177, *I* = 0.838) and PL and QHD populations (*D* = 0.025, *I* = 0.975) (Table 6). AMOVA analysis revealed that only 4% of genetic variation was partitioned among populations while 96% of the variation was concentrated within populations (Table 7).

**Table 5:** Genetic differentiation coefficient (*Fst*, below diagonal) and gene flow (*Nm*, above diagonal) among seven *P. trituberculatus* populations from the Bohai Sea.

Population	DL	DY	HW	HC	HLD	PL	QHD
DL		8.679	31.000	6.893	124.75	14.456	13.639
DY	0.028		7.103	3.917	8.371	6.000	5.564
HW	0.008	0.034		7.563	41.417	35.464	22.477
HC	0.035	0.060	0.032		8.083	12.908	13.639
HLD	0.002	0.029	0.006	0.030		18.981	16.417
PL	0.017	0.040	0.007	0.019	0.013		249.750
QHD	0.018	0.043	0.011	0.018	0.015	0.001

**Table 6:** Nei′s genetic distance (*D*, below diagonal) and genetic identity (*I*, above diagonal) among seven *P. trituberculatus* populations from the Bohai Sea.

Population	DL	DY	HW	HC	HLD	PL	QHD
DL		0.911	0.956	0.894	0.973	0.935	0.931
DY	0.094		0.895	0.838	0.908	0.879	0.872
HW	0.045	0.111		0.901	0.961	0.958	0.950
HC	0.112	0.177	0.104		0.906	0.934	0.937
HLD	0.028	0.097	0.040	0.099		0.945	0.939
PL	0.067	0.129	0.043	0.069	0.057		0.975
QHD	0.071	0.138	0.052	0.065	0.063	0.025

**Table 7:** Analysis of molecular variance (AMOVA) from seven *P. trituberculatus* populations.

Source of variation	*df*	*SS*	Variance component	Percentage of variation (%)
Among populations	6	656.921	1.302	4
Within populations	413	12,966.233	31.395	96
Total	419	13,623.155	32.697	100

*df* Degrees of freedom; *SS* Sum of squares.

## Discussion

Genetic diversity is a crucial criterion in estimating the adaptability of species to changing environments, hence a better understanding of the genetic diversity of species is vital for evaluating population structure and evolutionary dynamics^39^. Genetic diversity is susceptible to artificial selection, genetic drift, migration, and breeding systems^40^ and is normally evaluated by genetic parameters such as polymorphism information content (*PIC*), Shannon’s diversity index (*SI*), and heterozygosity (*H*). However, expected heterozygosity (*He*) could better reflect the genetic diversity of species than observed heterozygosity (*Ho*)^41^.

The current study reported *PIC* values of 40 SSR loci of 0.415 ~ 0.895, indicating the polymorphic nature of the loci and their suitability for assessing genetic diversity in the seven *P. trituberculatus* populations. Genetic analysis revealed that the genetic diversity of the wild populations (*He* ≥ 0.692) was higher than that of the cultivated population (*He* = 0.687), which was consistent with our previous report^42^. A similar result was found in *E. sinensis*^43^. In general, genetic drift, selection, and inbreeding resulted in low genetic variability in farmed stocks^44^. In addition, many SSR loci significantly deviated from HWE (*P* < 0.05), which might be attributed to null allele and heterozygote deficiency (*Fis* > 0). Null alleles might be accounted for insufficient sampling^45^ and variation of microsatellite flanking sequence^46^. Loss of heterozygosity might be accounted for migration, artificial selection, and inbreeding^47,48^, which was common in marine species such as *Scylla paramamosain*^49–51^, *Pinctada margaritifera*^52^, and *Hypophthalmichthys nobilis*^53^. Chen et al.^54^ used ten SSRs to investigate the effect of artificial selection on the genetic structure of two abalone lines and found a loss of heterozygosity (*Ho* = 0.650 < *He* = 0.711). These studies indicated the negative impact of heterozygote deficiency on population genetic diversity. Therefore, it is necessary to maintain a high level of genetic diversity in aquatic animals to reduce heterozygous loss and prevent germplasm degradation.

In terms of expected heterozygosity, this study showed lower genetic diversity of *P. trituberculatus* in the Bohai Sea (*He* = 0.725) than that in the Yellow Sea^47^ (*He* = 0.814) and the East China Sea^55^ (*He* = 0.916), which was consistent with the results revealed by SNP markers^14^. It has been shown that when conducting genetic diversity analysis on aquatic animals, the number of SSR loci should be greater than 20 and the sample size should be greater than 45^56^. The number of loci and sample size in this study meet this standard, indicating the reliable result of low genetic diversity of swimming crabs in the Bohai Sea. Bohai Sea is a semi-enclosed and shallow body of water that limits the dispersal of *P. trituberculatus*, leading to a decline in genetic diversity^47^. In the SSR investigation of *Exopalaemon carinicauda*, Zhang et al.^57^ suggested that the Binzhou population in the Bohai Sea had the lowest level of genetic diversity, which illustrated that the Bohai Sea might hinder the gene flow. Moreover, marine pollution, aquaculture pollution, and reclamation also reduced genetic diversity^58^. Therefore, it is necessary to carry out long-term genetic monitoring of *P. trituberculatus* in the Bohai Sea for full protection and utilization of the germplasm resources of this species.

A stable genetic structure is central to the survival of a species. Its disintegration leads to a reduction or even extinction of the population. Given the economic significance of *P. trituberculatus*, genetic monitoring of population structure is essential for the development of effective management strategies^13^. The results of the current study established that all *P. trituberculatus* individuals were divided into four subpopulations (Fig. 2). DY population indicated relatively low gene flow with other populations, which might be related to its geographical location. Dongying is located in the relatively closed Laizhou Bay, which restricts the gene exchange of *P. trituberculatus* with other populations in the Bohai Sea. The phylogenetic tree proved this result. The individuals from the HC population were located at the different clades in the phylogenetic tree, which illuminated a strong genetic mixing between cultured and wild individuals. It is speculated that the frequent gene flow between cultured and wild populations resulted from releases and artificial breeding by catching wild crabs as parents. For example, different regions shared the juvenile crabs of a full sibling family from the Huanghua farm for artificial breeding and releases, resulting in gene flow between the HC population and different wild populations. Therefore, formulating reasonable management measures is necessary to monitor the impact of the releases on wild populations and maintain the genetic integrity of cultivated populations. However, the phylogenetic tree was quite different from the PCA results, which might be due to the indistinct genetic differentiation and the close genetic distance between individuals. Additionally, the calculation methods between the phylogenetic tree and PCA analysis are different^59,60^. Further research is needed into the reasons for this difference.

The genetic differentiation index (*Fst*), an essential gauge of genetic differentiation among populations, is crucial to understand genetic relationships. 0 < *Fst* < 0.05, 0.05 < *Fst* < 0.15, 0.15 < *Fst* < 0.25, and *Fst* > 0.25 showed negligible, moderate, high, and strong genetic differentiation respectively^61^. In this study, HC and DY populations were medium differentiation (*Fst* = 0.060 > 0.05), which might be related to the geographical location of the two groups. Huanghua and Dongying were located at Bohai Bay and Laizhou Bay on both sides of the Yellow River estuary, respectively. The ecological environment, species distribution, and organic pollution in the Yellow River estuary led to the geographical differences between the two different sea areas^62,63^, which led to the differences in activity scope and habitat preference of *P. trituberculatus*, and ultimately resulted in high genetic differentiation between the HC and DY populations. In addition, geographic isolation also leads to low gene exchange between cultivated and wild populations compared to wild populations in the open sea, which can be proven by the genetic differentiation index. The average value of *Fst* between the HC and wild populations was 0.031, and between wild populations was 0.017 (Table 5). Moreover, the average value of gene flow (*Nm* = 31.289), genetic distance (*D* = 0.08), and genetic identity (*I* = 0.924) also demonstrated low genetic differentiation and strong genetic admixture among the seven *P. trituberculatus* populations.

## Conclusions

In summary, this study provided useful insights into the population structure of *P. trituberculatus* throughout the coastal areas of the Bohai Sea. Forty microsatellite loci revealed a low level of genetic diversity in the seven *P. trituberculatus* populations in the Bohai Sea. A low level of genetic differentiation and frequent gene flow among these seven populations were revealed, suggesting high genetic connectivity. The structure analysis illustrated four subpopulations, but the clustering pattern was not related to geographical location. To increase the genetic diversity of *P. trituberculatus*, practical and effective protective measures are expected to be taken to prevent the degeneration of germplasm resources. This study also provides a theoretical basis for selecting parents from different geographical populations during the artificial breeding programs.

## Data Availability

All data generated or analyzed during this study are included in this article.
